# Use of artificial intelligence in building personal branding and intercultural leadership

**DOI:** 10.3389/frai.2026.1885655

**Published:** 2026-08-10

**Authors:** Omer Cruz Caro, Doris Tarrillo Perez, Heily Consepcion Portocarrero Ramos, Yuri Reina Marín, Judith Nathaly Alva Tuesta, Jonathan Alberto Campos Trigoso, River Chávez Santos

**Affiliations:** 1 Oficina de Gestión de la Calidad, Universidad Nacional Toribio Rodríguez de Mendoza de Amazonas, Chachapoyas, Peru; 2 Facultad de Educación y Ciencias de la Comunicación, Universidad Nacional Toribio Rodríguez de Mendoza de Amazonas, Chachapoyas, Peru; 3 Unidad Funcional de Seguimiento al Egresado e Inserción Laboral, Universidad Nacional Toribio Rodríguez de Mendoza de Amazonas, Chachapoyas, Peru; 4 Facultad de Ingeniería Zootecnista, Biotecnología, Agronegocios y Ciencia de Datos, Universidad Nacional Toribio Rodríguez de Mendoza de Amazonas, Chachapoyas, Peru

**Keywords:** digital identity, intercultural competencies, professional visibility, technological appropriation, university graduates

## Abstract

The integration of AI tools reshaping how professionals learn, build reputation, and project their value in digitally and culturally diverse environments. Therefore, this study aims to analyze the relationships among AI tool use, personal branding, and leadership in intercultural contexts. A quantitative, non-experimental, cross-sectional, and correlational study was conducted with 169 university graduates from Peru, Ecuador, and Mexico. Data were collected using a Likert-scale questionnaire and analyzed through descriptive analysis, exploratory factor analysis, principal component analysis (PCA), and K-means clustering. The results indicate that 89.3% reported familiarity with AI tools, even though 79.3% had not received specialized training. Two profiles were also identified: one with moderate familiarity and another with greater technological appropriation, professional visibility, and intercultural competence. The PCA showed that the first two components explained 47.7% of the variance, with intercultural leadership, professional visibility, and technology adoption forming the central dimensions of the model. The results suggest that the use of artificial intelligence tools is associated with better career prospects when accompanied by critical judgment, authenticity, and cultural sensitivity. They also highlight the need to develop digital, ethical, and intercultural competencies in universities and organizations in order to guide relevant and responsible professional development processes.

## 1 Introduction

Artificial intelligence (AI) has gained significant influence in various fields, including academic, organizational, and professional fields (Dwivedi et al., 2021), and has transformed the way people use information, make decisions, and develop their identity in digital spaces (Caicedo et al., 2025; Dwivedi et al., 2021; Lara et al., 2023; Mikalef and Gupta, 2021). Furthermore, AI is increasingly being incorporated into talent management and professional communication (Huang and Rust, 2021b). Its technical aspects, based on predictive analytics, natural language processing, and machine learning, are reshaping how individuals define visibility, reputation, and strategic positioning (Chatterjee and Bhattacharjee, 2020; Huang and Rust, 2021a).

The current understanding of AI has shifted from a technical approach to a social and organizational perspective focused on the interaction between people and intelligent systems (Benbya et al., 2020; Mikalef and Gupta, 2021; Pappas et al., 2023; Van Meeteren et al., 2022). This transition suggests that the use of AI tools depends not only on technical skills but also on other factors such as training, trust, regulation, and organizational context (Van Meeteren et al., 2022; Zhang and Aslan, 2021). In this sense, professionals face the challenge of incorporating these technologies if they want to strengthen their presence, distinction, and value proposition, while managing ethical and structural uncertainties linked to their application (Guzman and Lewis, 2020).

AI has applications in professional development, such as content creation, data analysis, personalized communication, and decision-making support, helping individuals improve their reputation, capabilities, and how they are perceived by others in digital and multicultural environments (Huang and Rust, 2021b; Makarius et al., 2020). In turn, machine learning and natural language processing technologies contribute to better management of different audiences and increase positioning opportunities (Dwivedi et al., 2021). However, its use requires ethical implementation (Reina et al., 2025) because its advantages depend on maintaining a balance between cultural sensitivity, trust, authenticity, and efficiency (Arsenyan and Mirowska, 2021; Sarstedt et al., 2024).

From the perspective of technological acceptance, models such as the Unified Theory of Technology Acceptance and Use 2 (UTAUT2) allow us to understand that the adoption of AI in the workplace depends on social influence, perceived usefulness, enabling conditions, and effort expectancy (Tamilmani et al., 2021; Xue et al., 2024). In contexts where professional identity develops in digital spaces, transparency, authenticity, and trust in algorithms take on essential importance (Kelly et al., 2023). While the use of AI can improve communication, this type of technology can also create doubts about personal credibility and consistency (Kelly et al., 2023; Li et al., 2024; Shamim et al., 2023).

Personal branding is a strategic professional asset aimed at the intentional creation of identity, distinction, authenticity, reputation, and value proposition in digital ecosystems and labor markets (Gorbatov et al., 2018; Pham and Le, 2026; Szántó et al., 2025). Thus, the online identity that is projected can be strengthened or challenged in relation to others on professional networks such as LinkedIn or ResearchGate (Ilies et al., 2024; Pham and Le, 2026). Furthermore, the digital analytics tools and AI used in this process are transforming the way professionals create, communicate, and develop this identity, leading to greater access and visibility while also increasing the risk associated with a lack of authenticity (Arsenyan and Mirowska, 2021; Buhalis et al., 2023; Flavián et al., 2024).

Personal branding and intercultural leadership converge on three theoretical grounds that justify their integration into a single analytical model. First, both constructs treat authenticity as a precondition for credibility: personal branding scholarship identifies authenticity as central to trustworthy self-presentation (Gorbatov et al., 2018), while cultural-intelligence research treats it as foundational to trust across cultural boundaries (Sternberg et al., 2021). Second, both are externally validated rather than self-assessed, since personal branding derives its value from perceived employability and recognition by professional networks (Pham and Le, 2026), just as inclusive leadership is enacted only through followers’ evaluations of a leader’s behavior (Roberson and Perry, 2022). Third, both operate through a relational logic in which meaning is realized through others’ perceptions rather than internal attributes alone (Ashikali et al., 2021; Randel et al., 2018; Roberson and Perry, 2022). This convergence supports the relevance of personal branding as a theoretically grounded construct for examining its association with AI-supported self-presentation and perceived intercultural leadership legitimacy (Dwivedi et al., 2021).

Intercultural leadership in globalized contexts goes beyond adapting to diversity and cultural intelligence; it requires projecting an ethically coherent and culturally sensitive identity (Davaei et al., 2022; Earley, 2002; Sternberg et al., 2021). In this context, personal branding fulfills a symbolic validity function, as it facilitates the communication of credibility, cultural sensitivity, and the ability to influence diverse audiences (Gorbatov et al., 2018). Therefore, the strategic creation of a personal brand can be understood as a resource associated with intercultural leadership, trust in relationships, and professional visibility (Ashikali et al., 2021; Randel et al., 2018; Roberson and Perry, 2022).

AI can help in building a personal brand because these technologies can enhance visibility, positioning, and strategic success (Guzman and Lewis, 2020; Kim et al., 2024). However, they also raise a significant question about the authenticity, originality, and consistency of users’ identities. This dilemma is exacerbated, it is argued, in intercultural contexts where notions of trust, ethics, and cultural sensitivity require legitimate leadership practices (Bueechl et al., 2023). The adoption of AI, in this sense, is not only a technological opportunity but also a profound strategic challenge that must be addressed to maintain a solid professional identity in diverse intercultural environments (ÓhÉigeartaigh et al., 2020).

Despite the fact that the adoption of AI, digital communication, intercultural leadership, and personal branding has been explored in the literature, these topics have generally been examined separately (Benbya et al., 2020; Gorbatov et al., 2018; Mikalef and Gupta, 2021). There is little evidence on how the use of AI tools is associated with both personal branding and leadership projection in intercultural contexts (Dwivedi et al., 2021; Guzman and Lewis, 2020). This is because most research analyzes these phenomena in isolation, without sufficiently examining how AI adoption, personal branding, and professional legitimacy are associated in culturally diverse contexts.

To address this gap, this study proposes integrating AI adoption, personal branding, and intercultural leadership into a single analytical model. Its purpose is to examine how the use of AI tools is associated with personal branding and intercultural leadership within digitally and culturally diverse professional contexts. The research expands the understanding of personal branding as a relevant construct in the study of leadership and technology. In practical terms, it offers implications for professionals, educational institutions, multicultural organizations, and talent development programs.

Accordingly, this study examines the relationships among the use of AI tools, personal branding, and intercultural leadership among professionals operating in digital and multicultural environments. This approach allows for the joint analysis, through an analytical model, of topics that were previously considered individually, such as AI, personal branding, and intercultural leadership. This approach suggests how organizations and professionals may use AI appropriately for professional development purposes without losing their cultural identity. Furthermore, this research is crucial because very few studies in the region have explored these variables to date.

## 2 Methodology

### 2.1 Research design

The study was conducted using a quantitative, non-experimental, cross-sectional, and correlational approach. This design made it possible to examine associations among AI-tool use, personal branding, and intercultural leadership among university graduates working in diverse digital, professional, and cultural contexts.

Furthermore, the study adopted an analytical model in which AI tool use, personal branding, and intercultural leadership were examined as interrelated constructs within the professional and intercultural context of the participants. This structure allowed for the analysis of the associations among the three constructs, without assuming causal or mediating effects.

### 2.2 Study population

The study population consisted of university graduates from Peru, Ecuador, and Mexico, countries that share processes of digital transformation in higher education and the labor market, but which also exhibit sociocultural differences relevant to the analysis of intercultural leadership. This population was chosen because university graduates are at a key stage of professional transition, entry, or consolidation, in which building a digital identity, professional reputation, and the strategic use of emerging technologies become especially important.

The sample consisted of university graduates who voluntarily agreed to participate in the study and who met the following inclusion criteria: having completed university studies, residing in or coming from one of the countries considered, using or having knowledge of AI tools in academic, work, or professional contexts, and having experience in digital communication or professional positioning environments. Incomplete, duplicate, or inconsistent response patterns were excluded. A final sample of 169 participants was obtained.

The sampling method was non-probabilistic and based on convenience, due to the difficulty of obtaining a single sampling frame of university graduates from the three countries. However, efforts were made to ensure diversity in terms of national origin, field of study, and professional experience, with the aim of obtaining a broader understanding of the phenomenon within the Latin American context.

### 2.3 Data collection

The information was collected using a structured questionnaire, designed based on studies of AI adoption, personal branding, digital communication, and intercultural leadership. The instrument was organized into four sections: sociodemographic and professional data, use of AI tools, personal branding, and intercultural leadership.

The items were designed on a five-point Likert scale. This scale allowed us to capture the intensity of the participants’ perceptions and practices regarding the use of AI, their professional presence on digital platforms, their confidence in the results generated by these tools, the authenticity of their personal brand, and their ability to communicate, adapt, and lead in culturally diverse contexts.

The AI use variable included indicators related to frequency of use, trust in the tools, fields of application, perceived barriers, productivity, content creation, and support in professional communication. Personal branding variable considered aspects such as presence on digital platforms, value proposition, networking, recognition, visibility of achievements, and generation of professional opportunities. Finally, the intercultural leadership variable addressed dimensions related to cultural adaptation, active listening, valuing diversity, intercultural communication, creating inclusive environments, and leadership skills in diverse settings.

Table 1 describes the theoretical dimensions in terms of indicators and the operationalization of the instrument, relating each dimension to the items that comprise it and to the theoretical framework, based on studies.

**Table 1:** Operationalization of theoretical dimensions.

Dimension	Constituent items	Theoretical basis
AI adoption	Frequent AI use, trust use AI, field use, barriers, AI productivity, confidence in results	UTAUT2 (Tamilmani et al., 2021)
Digital personal branding	Presence platforms, value proposition, AI creation, personal branding, networking, brand recognition, presence achievements, brand opportunities	Gorbatov et al. (2018)
Intercultural leadership	Ease of adaptation, listening and valuing perspectives, communication form, leadership capacity, AI communication, inclusive environment, intercultural leadership	Sternberg et al. (2021); Ashikali et al. (2021)

Before final implementation, the instrument underwent content review to verify the clarity, relevance, and coherence of the items with the study variables. This review allowed adjustments to the wording of some questions to ensure they were understandable to participants from different countries and to avoid overly localized or ambiguous expressions.

### 2.4 Data collection procedure

Data were collected via a digital form, allowing graduates from different regions of Peru, Ecuador, and Mexico to participate. Before beginning the questionnaire, the objectives of the study, the voluntary nature of participation, the confidentiality of the information, and the exclusively academic use of the data were briefly explained.

Informed consent was obtained from participants before they completed the survey. No sensitive data that would allow for the direct identification of respondents was requested, and the information was processed in aggregate form. This procedure protected the privacy of participants and ensured the ethical handling of the information.

### 2.5 Data analysis

The data were organized, cleaned, and coded in a statistical database, retaining only valid cases for analysis. Before processing the data, a consistency analysis was performed to detect incomplete responses, outliers, and data redundancy, which could affect data quality. A descriptive analysis was conducted on the sociodemographic variables and questionnaire items, including frequencies, percentages, means, and standard deviations.

**Figure 1:**
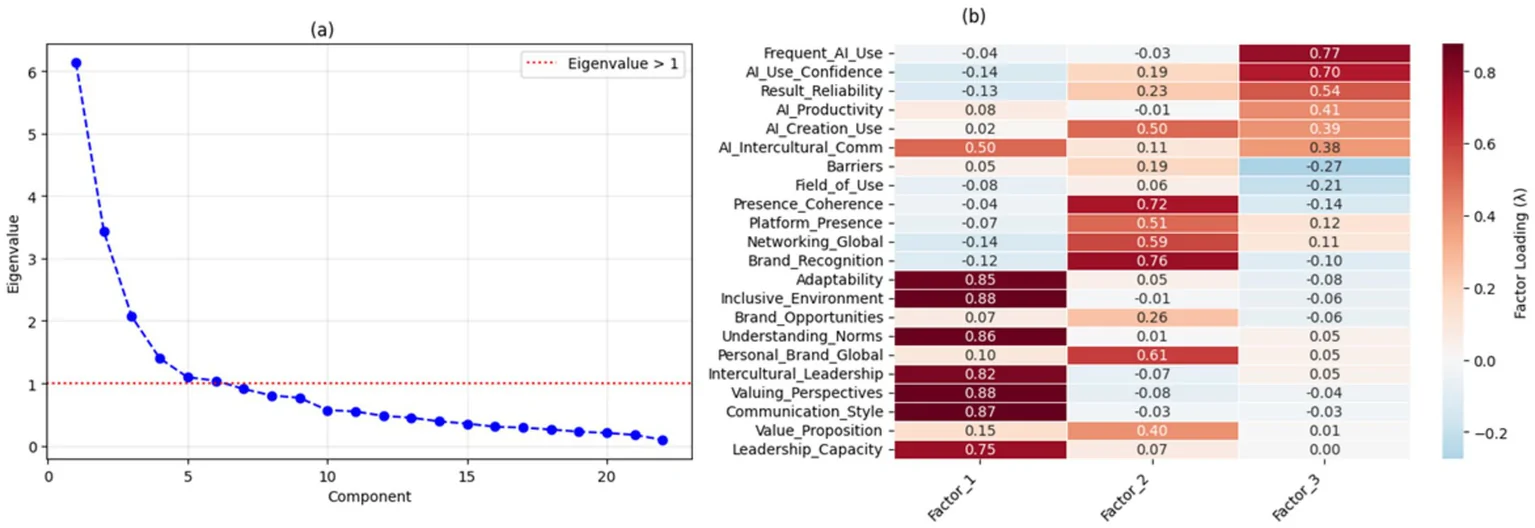
Exploratory factor analysis of the instrument: Scree plot and factor loading matrix with Promax rotation. The factor structure confirms the three proposed dimensions. Factor 1 groups items related to intercultural leadership; Factor 2 groups indicators related to digital personal branding; and Factor 3 groups items associated with AI adoption. The total variance explained by the three factors was 45.98%, distributed as follows: 24.45% for Factor 1, 12.28% for Factor 2, and 9.25% for Factor 3. Factor loadings with |*λ*| ≥ 0.40 were considered significant. **(a)** presents the scree plot, which allows observation of the distribution of eigenvalues of the extracted components. **(b)** shows the factor loading matrix obtained by Promax rotation.

To explore the internal structure of the instrument, an Exploratory Factor Analysis (EFA) was carried out on a valid sample of 169 observations and 22 variables (Figure 1). This procedure was intended to establish how, through empirical groupings, the items clustered and whether this structure was consistent with the proposed theoretical dimensions: adoption of AI, digital personal branding, and intercultural leadership. The complete questionnaire and detailed validation process, including the item structure, adequacy tests, factor loadings, explained variance, and reliability analysis, are provided in Supplementary material S1.

The data were assessed for appropriate factor analysis using Bartlett’s test of sphericity and the Kaiser-Meyer-Olkin (KMO) index before performing the factor analysis. Bartlett’s test was statistically significant (*χ*^2^ = 1910.97; *p* = 7.8381*e* − 262), suggesting sufficient correlation between the items. In fact, the overall KMO index was 0.801, an acceptable value as it indicates that the correlation matrix provides a suitable framework for factor reduction.

The EFA was conducted using a three-factor solution, in accordance with the instrument’s conceptual structure and the interpretation of the scree plot. To improve the interpretation of the factors, Promax rotation was used, as it allows for the assumption of correlations between the analyzed dimensions, which is relevant in social and educational studies where constructs may be related to one another. Factor loadings were considered relevant when their absolute values were equal to or greater than 0.40 (|*λ*| ≥ 0.40).

The data showed that Factor 1 primarily encompassed items related to Intercultural Leadership, such as an inclusive environment, understanding of norms, valuing perspectives, communication style, intercultural leadership, and leadership skills. Factor 2, meanwhile, concentrated on items associated with Digital Personal Branding, for example, consistency of presence, presence on platforms, global networks, brand recognition, and global personal branding. Finally, Factor 3 grouped indicators linked to AI Adoption, such as frequent use of AI, confidence in its use, reliability of results, and productivity thanks to AI.

Regarding the explained variance, Factor 1 accounted for 24.45% of the total variance; Factor 2, 12.28%; and Factor 3, 9.25%. These three factors represented 45.98% of the cumulative variance, an acceptable level in exploratory social science studies, given that constructs are often influenced by multiple latent dimensions.

Based on the obtained factor structure, the internal consistency of the constructs was analyzed using Cronbach’s alpha coefficient. The results were adequate for AI adoption (*α* = 0.740) and digital personal branding (*α* = 0.789), and excellent for intercultural leadership (*α* = 0.942). These results demonstrate that the items exhibit appropriate internal coherence and that the instrument has a factor structure that is interpretable, consistent, and aligned with the proposed theoretical dimensions.

Following this, principal component analysis (PCA) was used to synthesize the information contained in the items and represent the contribution of the components to the total variance. This procedure allowed for observing the distribution of the indicators in a limited space, analyzing the variance explained by each component, and visualizing the relationship between the variables through the correlation circle. For interpretation, component loadings, communalities, and individual and cumulative variance were considered. Thus, PCA was used as a technique for representing and synthesizing the empirical structure of the data, not as a substitute for the previously applied factor analysis.

Additionally, a cluster analysis was conducted using the K-means algorithm to identify distinct participant profiles based on their scores in AI adoption, personal branding, and intercultural leadership. This procedure allowed participants to be classified into two groups or clusters, differentiated by their levels of technological integration, professional positioning, and intercultural competence. The clusters were represented using principal component projection, incorporating centroids and confidence ellipses to visualize the separation between the groups.

The two-cluster solution was retained based on the interpretability of the profiles, the separation of centroids observed in the PCA projection, and the coherence of the resulting groups with the theoretical dimensions of AI adoption, personal branding, and intercultural leadership. However, future studies should complement this decision with additional clustering diagnostics, such as the elbow method, silhouette coefficient, Calinski-Harabasz index, or Davies-Bouldin index.

Cluster correlation matrices were developed to analyze how the indicators are internally related within each profile. This analysis allowed for a comparison of the association patterns between AI adoption, trust in its use, productivity, personal branding, networking, recognition, intercultural communication, and leadership. The purpose of this procedure was to identify whether the relationships between the variables behaved similarly or differently in each cluster.

All results were presented using figures and tables, including descriptive figures, scree plot of the factor analysis, segmentation using K-means, cluster correlation matrices, component and communality matrix, variance explained by principal components, and PCA correlation circle.

## 3 Results

**Figure 2:**
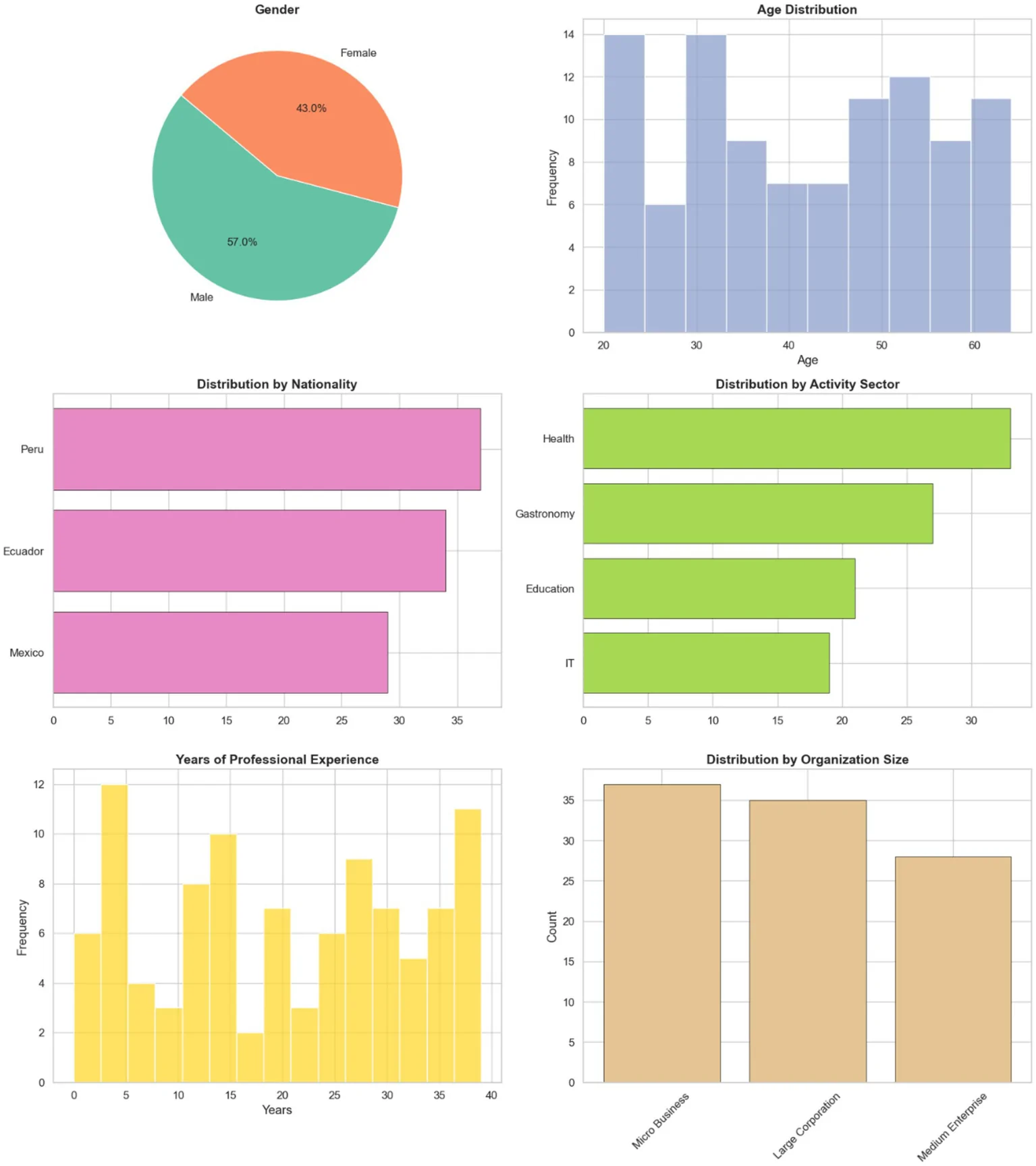
Sociodemographic and occupational characteristics of the participants.

The sociodemographic and occupational profile of the participants is shown in Figure 2. Regarding gender, the sample was relatively balanced, with a slight male predominance (54.0%) compared to female participation (46.0%). Regarding age, younger respondents, older individuals, and participants at different stages of professional development represented various levels of professional development.

In terms of country of origin, graduates from Ecuador, Peru, and Mexico participated: Ecuadorian graduates represented the largest group, followed by Peruvian and Mexican graduates. This composition allows the study to be contextualized within the Latin American sphere and to analyze variables across a variety of sociocultural realities.

Furthermore, the participants came from disciplines ranging from health, food, education, and information technology, among others. The diversity of professional fields supports the relevance of analyzing AI tools, personal branding, and intercultural leadership as cross-cutting phenomena. Their work experience revealed a wide range of respondents, from professionals with limited years of experience to participants with over three decades of experience. Participation from individuals associated with medium-sized companies was predominant, followed by micro-enterprises and large corporations, providing insight into the phenomenon across diverse organizational contexts.

**Figure 3:**
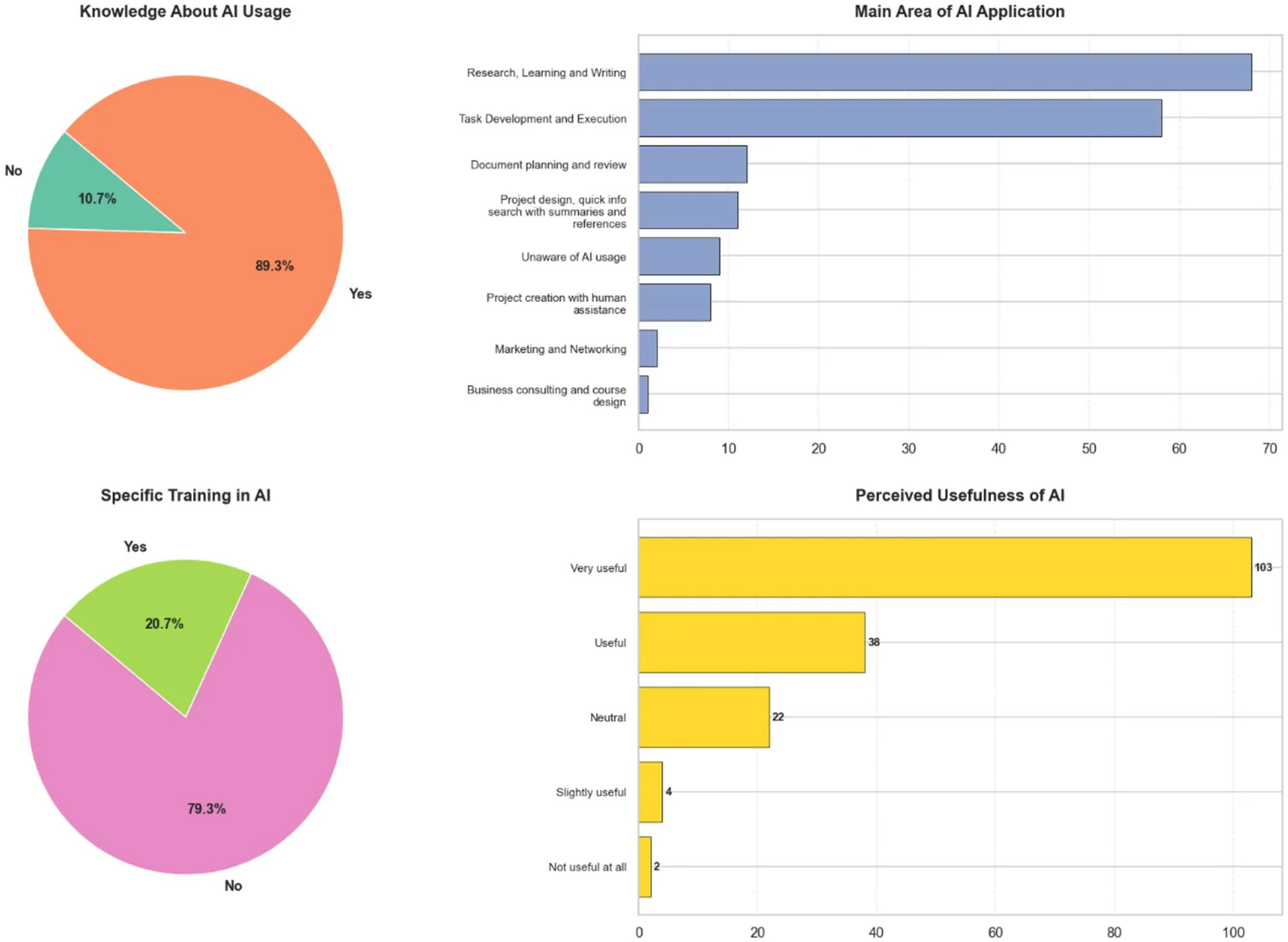
Profile of knowledge, training, application, and perceived usefulness of AI.

Figure 3 presents the profile of knowledge, training, application, and perceived usefulness of AI among the participants in this study. The findings reveal that 89.3% reported knowing how to use AI tools, while 10.7% do not. A significant gap was observed in specific training, with 79.3% indicating they had not received formal AI training, and only 20.7% reporting such training.

Regarding areas of use, AI was mainly used for research, learning, and writing, followed by task development and execution. It was reported to be used less frequently in document planning and review, project design, rapid information retrieval, human-assisted project creation, marketing, networking, and business consulting. These results indicate that AI is mainly used as an academic, cognitive, and productivity support tool.

Furthermore, the perceived usefulness was largely favorable. The majority of participants considered AI to be “very useful,” followed by those who rated it as “useful.” Negative assessments were few, suggesting a favorable perception of these tools within the professional and educational context analyzed. Overall, the results show that AI is recognized as a valuable resource, although its adoption is still developing primarily without sufficient specialized training.

**Figure 4:**
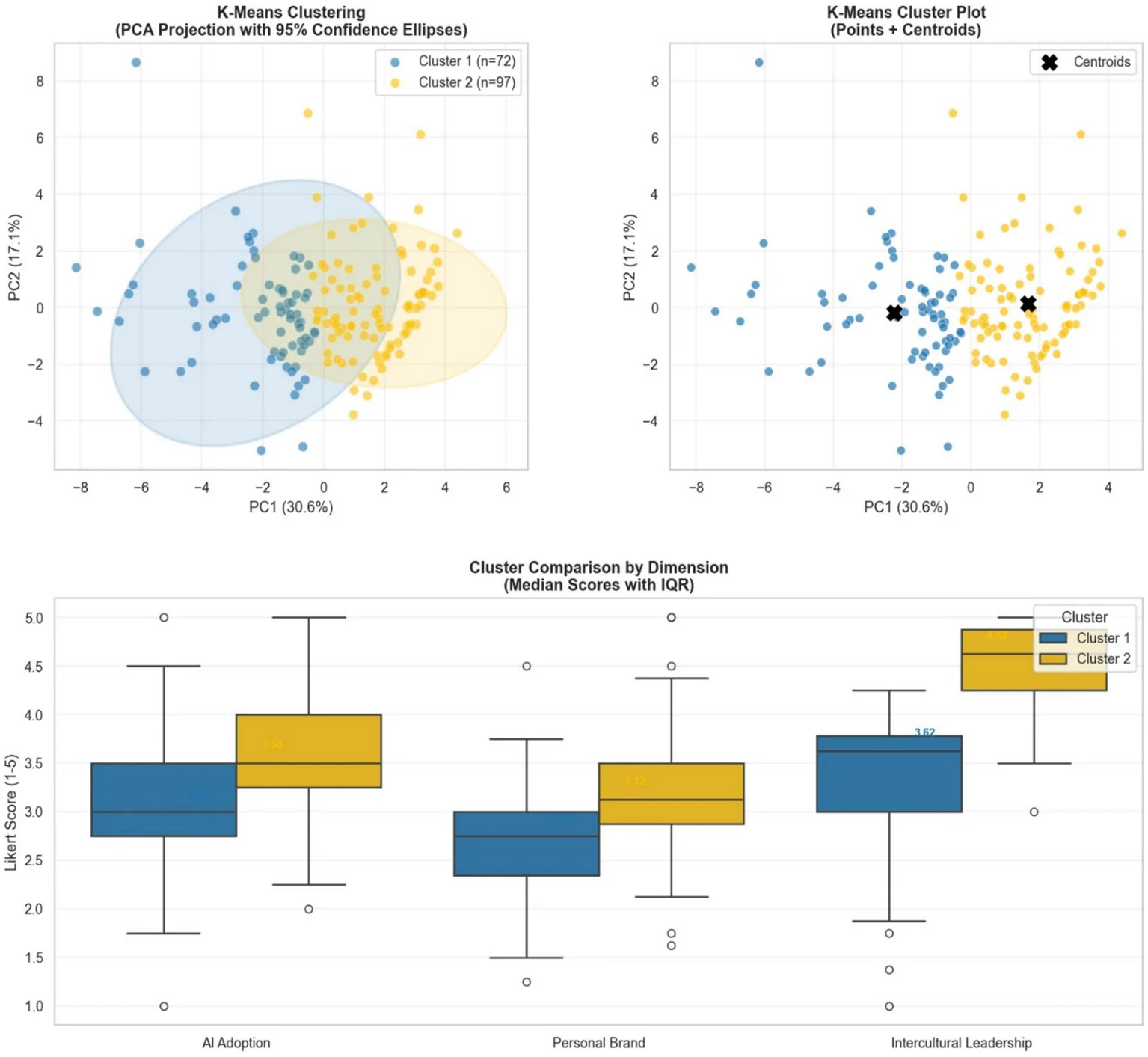
K-means clustering of participants based on AI adoption, personal branding, and intercultural leadership.

Figure 4 shows the segmentation of participants using the K-means algorithm, considering the dimensions of AI adoption, personal branding, and intercultural leadership. The analysis identified two distinct clusters: Cluster 1, consisting of 72 participants, and Cluster 2, consisting of 97 participants.

Principal component analysis shows that the first component explained 30.6% of the variance and the second component 17.1%, allowing visualization of the distribution of participants according to their profiles. Although some overlap is observed between the clusters, the centroids show an observable separation between the two groups, especially along the first principal component.

The comparative analysis by dimension shows that Cluster 2 obtained higher scores in AI adoption, personal branding, and intercultural leadership. The most notable difference was in intercultural leadership, where Cluster 2 showed a higher median score than Cluster 1. This indicates that participants with higher scores in AI adoption and personal branding also showed higher scores in intercultural leadership within the analyzed sample.

Cluster analysis identified a participant profile with higher scores in technological integration, professional positioning, and intercultural competence. These results suggest that AI adoption may be examined not only as a functional practice, but also as a dimension associated with personal branding and intercultural leadership within culturally diverse professional environments.

Table 2 presents the PCA component loadings and communalities of the individual variables. The variances are greater than 0.50 overall, indicating that the components adequately explain the variance of the items included in the instrument. This indicates that the components provide an accurate representation of the instrument’s structure, which is essential for ensuring the accuracy and reliability of the measurements taken.

**Table 2:** Principal component loadings and communalities for AI adoption, personal branding, and intercultural leadership indicators.

Variable	F1	F2	F3	F4	F5	F6	Communality
Frequent AI use	0.064	−0.008	0.786	−0.004	−0.288	−0.242	0.763
Trust use AI	−0.003	0.226	0.790	−0.054	−0.056	−0.049	0.684
Field use	−0.020	0.113	0.009	0.082	−0.150	0.794	0.673
Barriers	−0.023	−0.014	−0.043	−0.058	0.869	−0.157	0.786
AI productivity	0.137	−0.168	0.593	0.286	0.386	0.233	0.684
Confidence results	−0.020	0.185	0.720	0.129	0.209	0.240	0.672
Presence platforms	−0.010	0.344	0.196	0.661	−0.017	−0.199	0.633
Value proposition	0.208	0.252	0.109	0.546	0.012	0.300	0.507
AI creation	0.153	0.528	0.434	0.217	−0.160	−0.349	0.686
Personal branding	0.211	0.698	0.076	0.178	0.113	−0.177	0.614
Networking	−0.021	0.743	0.220	−0.101	0.053	0.057	0.618
Brand recognition	−0.002	0.779	0.034	0.099	0.000	0.276	0.693
Presence achievements	0.052	0.680	−0.048	0.285	−0.243	0.068	0.612
Brand opportunities	0.069	−0.002	−0.049	0.792	−0.031	0.075	0.641
Ease of adaptation	0.866	0.096	−0.032	0.042	−0.076	0.058	0.771
Listening and valuing perspectives	0.870	−0.060	−0.008	0.092	−0.005	0.061	0.772
Communication form	0.874	0.014	0.008	0.062	0.032	−0.010	0.769
Leadership capacity	0.805	0.172	0.028	−0.044	0.064	0.061	0.688
AI communication	0.606	0.255	0.367	−0.049	−0.050	−0.317	0.672
Inclusive environment	0.873	0.018	−0.032	0.088	−0.052	−0.052	0.777
Intercultural leadership	0.839	−0.032	0.078	0.056	0.041	−0.043	0.717

The first component mainly grouped indicators related to intercultural leadership. The highest loadings were in adaptability (0.866), listening and appreciation (0.870), communication style (0.874), leadership capacity (0.805), inclusive environment (0.873), and intercultural leadership (0.839). These findings indicate that intercultural leadership is the strongest component of the instrument and shows good consistency across its indicators.

The second component focused heavily on personal branding, specifically on brand recognition (0.779), networking (0.743), personal branding (0.698), and achievement presence (0.680). Content is also positively associated with AI-supported content creation (0.528), suggesting an interconnection between personal branding, digital visibility strategies, professional positioning, and the effective use of digital tools.

The third component consisted primarily of indicators of adoption and trust in AI. Key indicators in this component included frequent use of AI (0.786), trust in the use of AI (0.790), trust in the results (0.720), and AI for productivity (0.593). This result allows us to interpret this component as a dimension associated with the practical appropriation of AI, where frequent use is related to trust in the tool and the results it produces.

The fourth component was linked to professional visibility and the opportunities generated by personal branding. The highest scores were observed in branding and opportunities (0.792), presence on digital platforms (0.661), and value proposition (0.546). This indicates that this component expresses a dimension oriented toward the professional’s external positioning, especially in digital spaces.

The fifth component was primarily represented by the barriers indicator (0.869). This shows that the perceived difficulties in using AI constitute a distinct dimension within the model, likely associated with limitations in access, training, trust, technical expertise, or institutional conditions.

The sixth component was primarily associated with the field of AI use (0.794). This result indicates that the specific area where AI is applied constitutes another relevant dimension, given that AI use can vary depending on the type of professional, academic, communicational, or strategic activity carried out by the participants.

**Figure 5:**
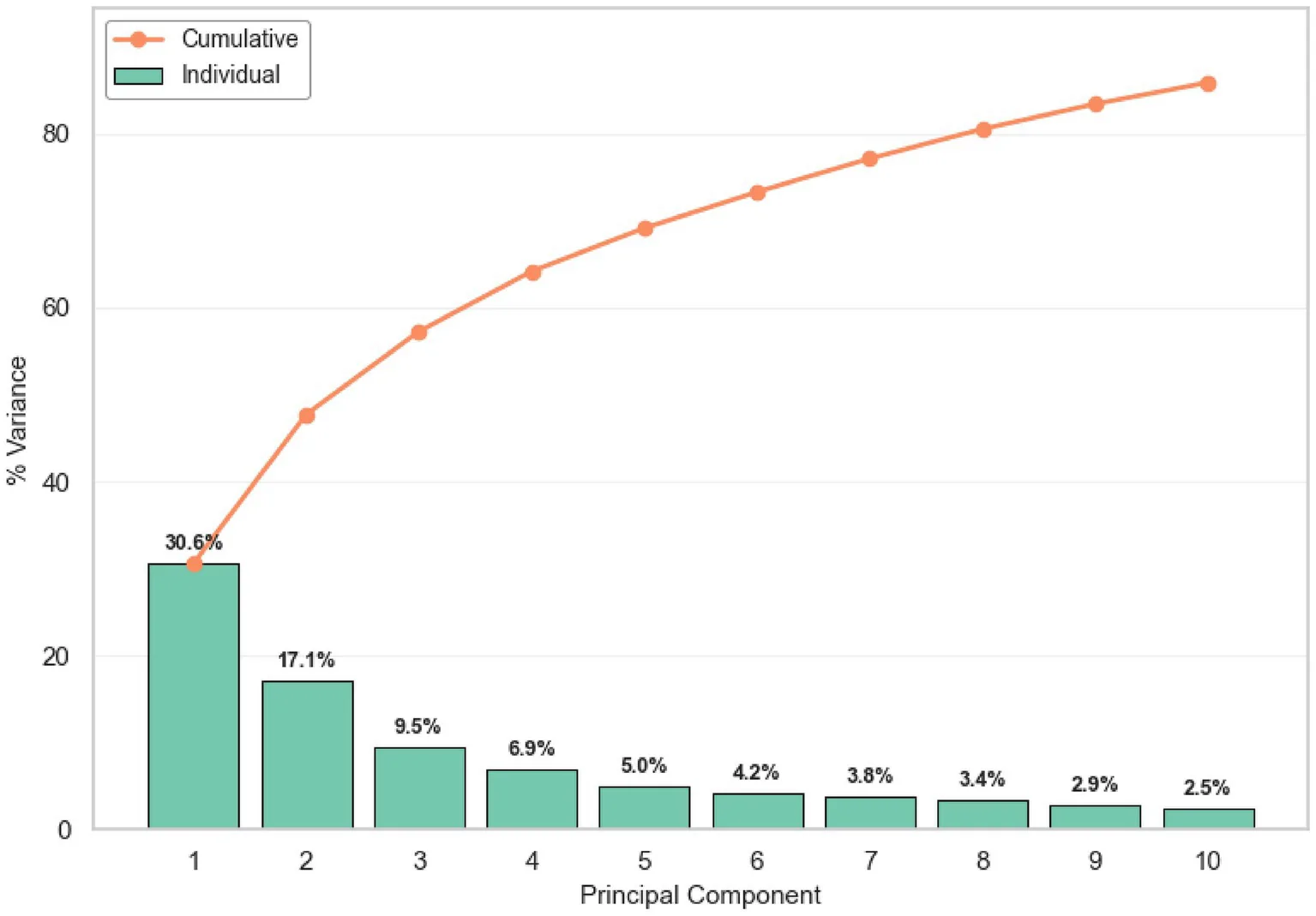
Explained variance and cumulative variance of the principal components.

Principal component analysis allowed us to synthesize the instrument’s internal structure and identify the components with the greatest explanatory power. As shown in Figure 5, the first component explained 30.6% of the total variance, and the second component explained 17.1%, together accounting for 47.7% of the information contained in the data. When considering the first six components, the cumulative variance reached approximately 73.3%, demonstrating a suitable solution for representing the instrument’s multidimensional structure.

**Figure 6:**
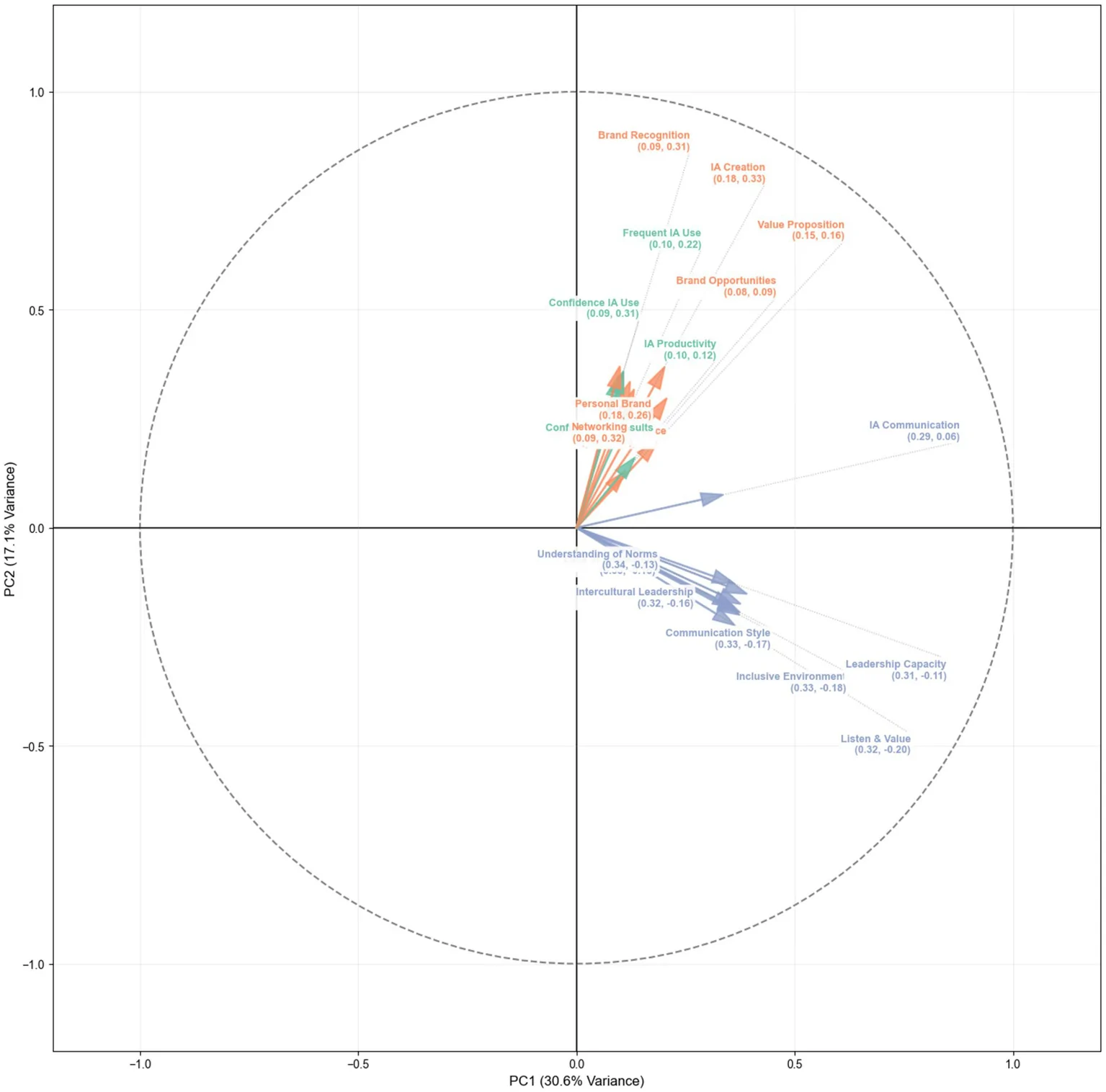
PCA correlation circle for AI adoption, personal branding, and intercultural leadership indicators.

Figure 6 shows the correlation circle for the first two principal components. The horizontal axis represents PC1, which explains 30.6% of the variance, while the vertical axis represents PC2, which explains 17.1%. Together, these two components allow us to visualize the orientation and association of the items within the reduced factor space.

The first principal component shows a clear concentration of indicators related to intercultural leadership, such as adaptability, communication style, leadership skills, inclusive environment, listening and appreciation, understanding of cultural norms, and intercultural leadership. These items are located primarily on the right side of the graph, indicating that they share a common direction and contribute significantly to the construction of the first component. This allows us to interpret PC1 as an axis primarily associated with intercultural competencies, inclusive communication, and leadership in diverse contexts.

The second principal component, meanwhile, more clearly differentiates between indicators related to personal branding and the strategic use of AI. Variables such as brand recognition, AI-powered content creation, value proposition, career opportunities, frequent AI use, and trust in AI use are located in the upper part of the plot. This suggests that PC2 captures a dimension more closely linked to professional visibility, digital positioning, and technological adoption.

The distribution of the vectors also shows that the personal branding and AI adoption indicators are oriented in a similar direction, suggesting a conceptual association between the use of technological tools and the construction of a visible professional identity. In contrast, the intercultural leadership indicators, while related to the overall model, exhibit a more distinct orientation, confirming that they constitute a separate dimension within the instrument’s structure.

**Figure 7:**
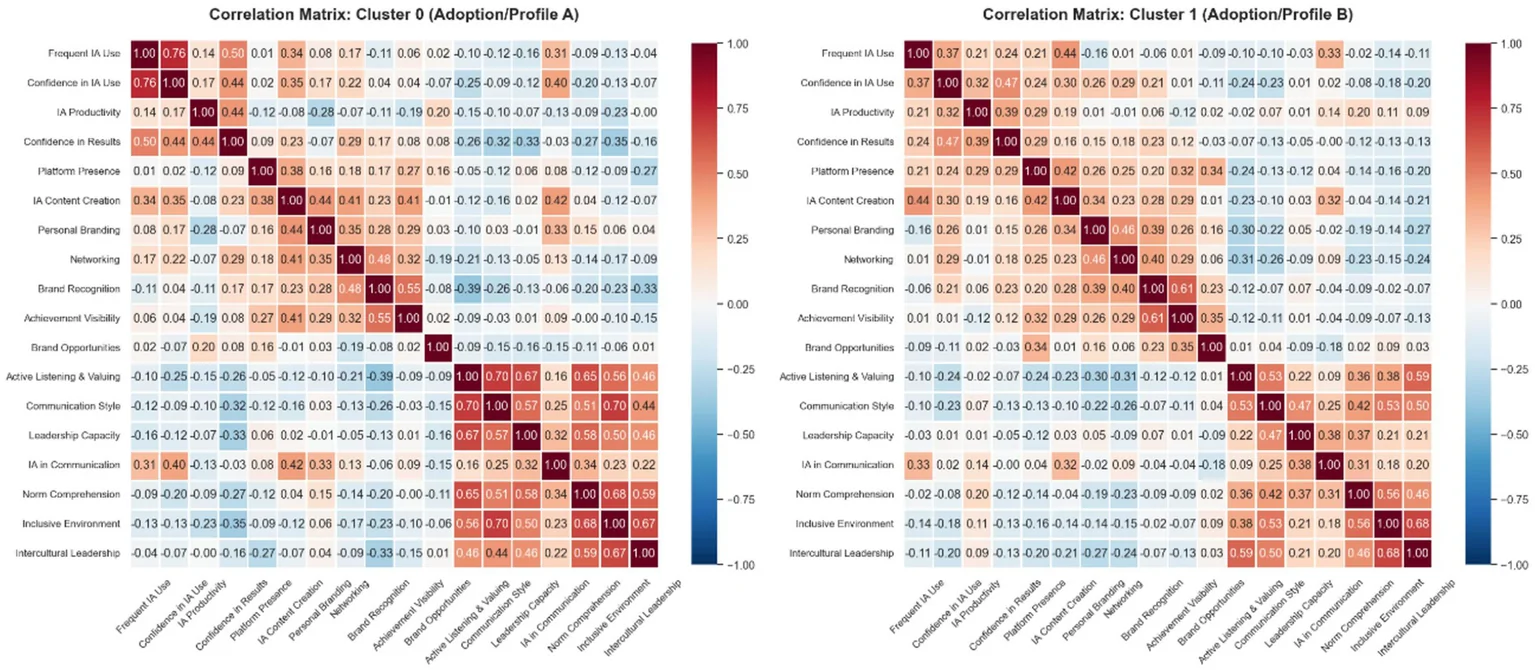
Correlation matrices by cluster for AI adoption, personal branding, and intercultural leadership indicators.

The correlations for the two groups were plotted in Figure 7. In Cluster 0 (or Profile A), there was a strong relationship between higher frequency and confidence in AI use, suggesting that hands-on experience with these tools may be associated with a greater perception of confidence in their use. Intercultural leadership markers also showed positive correlations, including listening, communication, cultural understanding, creating inclusive environments, and intercultural leadership.

In Cluster 1, or Profile B, the correlations between AI indicators were moderate but showed stronger articulation, with dimensions connected to productivity, personal branding, and career opportunities. Among the factors linked to personal branding in this group, networking, recognition, achievements, and opportunities were more consistently associated, indicating a more planned approach to professional visibility.

The findings indicate that each group has a unique relational pattern. Profile A shows a very strong positive relationship between the amount of AI used and self-confidence, but also reveals an internally integrated intercultural facet. Profile B shows a broader trend of convergence between technology application, productivity, self-presentation, and intercultural leadership. These results suggest that AI adoption does not develop uniformly among participants; rather, it is characterized by divergent behavioral patterns in terms of confidence, professional positioning, and intercultural competencies.

## 4 Discussion

According to the findings, the adoption of AI by university graduates in Peru, Ecuador, and Mexico is not merely technological but also a social, educational, and professional process. The sample includes participants from different countries, diverse productive sectors, ages, and organization sizes, but the sample suggests that AI is being adopted across a broader spectrum of professions, not just select niches. This is consistent with Dwivedi et al. (2021), Itodo et al. (2024), and Alt et al. (2020), who argue that AI transforms several aspects of human, organizational, and professional activity, in parallel with Benbya et al. (2020), Gorbatov et al. (2018), and Mikalef and Gupta (2021), that it is important to recognize that AI is a socio-technical capability that is affected by people, organizations, context, and strategic application.

One of the findings is that respondents had a high level of familiarity with using AI tools, with 89.3% indicating they were familiar with them. However, this finding contrasts with the lower level of specific training reported by respondents, as 79.3% reported having received no formal AI training. This gap reveals a crucial scenario for professionals who are adopting AI for academic, work, and communication purposes, but generally only on their own initiative, or through casual use of AI, driven by a work-related tendency or the immediate need to solve tasks. This is consistent with the assertion of Arsenyan and Mirowska (2021), Buhalis et al. (2023), and Flavián et al. (2024) that AI literacy should not be limited to the instrumental use of tools, but should also extend to conceptual understanding, critical evaluation, ethical implications, and responsible application. This resonates with Van Meeteren et al. (2022), Dai et al. (2011), and Zhang and Aslan (2021), noting that the incorporation of AI-based technologies also requires the formation of practice systems that go beyond the unplanned or superficial use of these tools, and instead offer a conscious pedagogical and professional appropriation.

The perception of AI was positive among users, with the majority saying it was “very useful” or “useful.” This finding supports theories of technology acceptance, particularly the Unified Theory of Technology Use and Acceptance 2 (UTAUT2), which posits that perceived usefulness, enabling conditions, and performance expectancy all impact the intention to use a technology (Tamilmani et al., 2021; Xue et al., 2024). For the present study, perceived usefulness is primarily expressed in research, learning, writing, task development, and document planning activities. This is consistent with Huang and Rust (2021b), who argue that AI amplifies human abilities by facilitating analysis, personalization, communication, and decision-making, which also raises concerns. However, the findings also raise warnings because a high perceived utility without specific training can magnify the risks of technology dependence, poor critical evaluation, loss of communicative authenticity, and unethical behavior in handling AI-generated results (Dwivedi et al., 2021; Guzman and Lewis, 2020).

The PCA showed how the indicators were organized within the structure of the collected data. The first component was mainly defined by intercultural leadership indicators, showing the strength of this dimension’s structural level. This information is relevant because it suggests that intercultural leadership functions as an integrated competency encompassing adaptation, active listening, communication, and the creation of inclusive spaces, all as part of the same relational logic. This is consistent with Ashikali et al. (2021), Randel et al. (2018), and (Roberson and Perry, 2022) who argue that inclusive leadership is strengthened when it promotes an opportunity for people to feel recognized, valued, and able to participate despite their cultural differences.

The second dimension of the PCA grouped personal branding indicators such as recognition, networks, achievements, and professional identity building. This reinforces the notion that personal branding serves as a relational and reputational framework in which professional visibility depends not only on a presence in digital channels, but rather on communicating a recognizable, coherent, and socially validated value proposition. This is consistent with Gorbatov et al. (2018), Giermindl et al. (2022), and Mirowska and Arsenyan (2025), who argue that personal branding is characterized by authenticity, differentiation, and proactive reputation management. Likewise, Arsenyan and Mirowska (2021) and Sarstedt et al. (2024) argue that digital visibility can increase the reach of professional identity; however, it can generate tensions around authenticity, credibility, and consistency between the person and the projected image.

The third component included indicators of AI adoption and trust, specifically frequent use, confidence in its use, productivity, and confidence in the results. This suggests that, from the participants’ perspective, AI adoption is based not only on access to the tool but also on the trust that develops from experience using it. The results are consistent with Huang and Rust (2021b), Gorbatov et al. (2018), and Szántó et al. (2025), who argue that human trust in AI is a determining factor for its effective integration into organizational and professional contexts. Furthermore, it aligns with Kelly et al. (2023), Li et al. (2024), and Shamim et al. (2023), who argue that the acceptance of intelligent systems depends not only on perceived usefulness but also on cognitive trust, transparency, and perceived reliability of the results.

K-means-based cluster analysis identified two distinct participant profiles. Cluster 1 participants showed more moderate levels in their adoption of AI, personal branding, and intercultural leadership, while Cluster 2 members showed higher values across all three dimensions. This finding is relevant because it suggests that AI adoption is not uniform; rather, there are professional profiles that more fully integrate technology, personal positioning, and cross-cultural skills. This finding aligns with Benbya et al. (2020); Mikalef and Gupta (2021); Pappas et al. (2023); Van Meeteren et al. (2022), who state that digital transformation is based on context, available capabilities, and organizational practices. This also aligns with Van Meeteren et al. (2022), who emphasize that responsible digitization needs to consider the differences between users, institutions, and social environments. Likewise, the qualitative findings of Aaker (1997) and Szántó (2025) participants consistently recognized attributes such as visibility, credibility, and differentiation as essential components of an effective personal brand. These attributes enhance an individual’s employability and strengthen their professional presence in competitive environments (Nur, 2023; Szántó et al., 2025).

The greatest variation among the clusters was observed in intercultural leadership, where Cluster 2 achieved higher levels. This suggests that, within the analyzed sample, participants with higher levels of technological integration and professional visibility also reported higher levels of intercultural leadership. This association may be related to the fact that the judicious use of AI is connected with communication practices, message adaptation for diverse audiences, access to multicultural information, and professional image management in digital spaces (Haddad, 2024; Klimova and Hua Chen, 2024; Sarwari et al., 2024). However, this connection should not be taken as an automatic or even deterministic relationship (ÓhÉigeartaigh et al., 2020; Wong, 2020). As ÓhÉigeartaigh et al. (2020) point out, ethical responsibility, cooperation, and recognition of cultural differences are essential when pursuing technological advancements in intercultural contexts. AI may be associated with intercultural leadership development only when it is approached with reason, reflection, and an appreciation for difference (Ahmad, 2025; Diaz, 2025).

Cluster correlation matrices also contribute to the analysis. In Profile A, there is a strong correlation between AI usage volume and confidence in its use, indicating that practical experience may be associated with a greater sense of confidence regarding AI tools. Conversely, in Profile B, the connections between AI, productivity, personal branding, and career prospects were more focused, suggesting a more calculated application of the technology. This result is consistent with Huang and Rust (2021b), where the integration of AI in organizations depends not only on the accessibility of the technology but also on how people incorporate it into their relevant activities, decisions, and practices. This is also corroborated by the arguments of Benbya et al. (2020), Mikalef and Gupta (2021), Pappas et al. (2023), and Van Meeteren et al. (2022), who argue that the capacity of AI is only articulated when there is a transformation of technological resources into organizational capabilities and concrete results.

The academic sphere, AI adoption, personal branding, and intercultural leadership are not isolated dimensions; on the contrary, they are interconnected processes in the professional trajectory of university graduates. While AI is useful in terms of productivity, communication, and visibility in the workplace, its greatest value lies in the user who is able to combine it with sound judgment, authenticity, and cultural sensitivity (Rinta-Kahila et al., 2021). This result is consistent with what has been suggested (Dwivedi et al., 2021; Guzman and Lewis, 2020). The study suggests that digital technologies are associated with communication practices and with the ways individuals build their reputation, trust, and professional identity. In this context, the study’s findings are consistent with a more cohesive personal brand being associated with stronger projection of intercultural leadership among participants in this study, especially when AI is leveraged as strategic support rather than as a substitute for human judgment. The limited formal training in AI observed among the participants suggests that universities should promote digital skills, technology ethics, and intercultural communication to prevent superficial, dependent, or uncritical use of these tools (Arsenyan and Mirowska, 2021; Sarstedt et al., 2024).

This study contributes to understanding how AI adoption, personal branding, and intercultural leadership are articulated among Latin American professionals, although it is not without limitations. Non-probabilistic convenience sampling, together with the heterogeneity of the sample in terms of country, professional field, and career stage, restricts the generalizability of the results to the broader population of Latin American university graduates. Likewise, the cross-sectional, non-experimental design based on self-reported data precludes establishing causal relationships and does not rule out common-method variance. Accordingly, these findings should be interpreted as preliminary, context-specific evidence, whose external validity requires confirmation through future studies with larger, more balanced, and representative samples.

This study offers an initial approach to understanding the associations among AI adoption, personal branding, and intercultural leadership within the specific context analyzed. These associations require further investigation, especially since AI-based professional practices are constantly evolving and their connections with professional identity, digital communication, and intercultural dynamics still require more empirical evidence and contextualized analysis.

## 5 Conclusion

The study suggests that AI is becoming relevant in the professional trajectories of the university graduates analyzed, not only as a technological tool but also as a resource associated with learning, communication, professional positioning, and digital identity construction. In this sense, AI adoption should be understood as a process linked to trust, perceived usefulness, training, ethical considerations, and the ability to integrate technology into meaningful professional practices.

The use of AI tools, personal branding, and intercultural leadership are interrelated, but the results must be interpreted from a descriptive and exploratory perspective. The responsible use of AI tools can lead to greater professional visibility and reputation. However, their value depends on authenticity, critical thinking, and sensitivity to cultural diversity.

Intercultural leadership emerges as a relevant competency in digital and culturally diverse environments. Its development is not limited to technical competence, but also involves human skills such as listening, adaptation, respectful communication, appreciation of different perspectives, and the creation of inclusive environments. Therefore, AI may support these processes, but it cannot replace human judgment, social experience, or contextual understanding.

From a practical perspective, the results suggest the need for universities, organizations, and professional training programs to promote AI training with an ethical, critical, and intercultural approach. It is not enough to provide training on the operational use of digital tools; it is also necessary to train professionals who can use them to support their performance, communicate responsibly, manage their personal brand, and participate effectively in intercultural and global environments.

This study offers an initial approach to understanding the associations among AI adoption, personal branding, and intercultural leadership within the specific context analyzed. These associations require further investigation, especially since AI-based professional practices are constantly evolving and their connections with professional identity, digital communication, and intercultural dynamics still require broader empirical evidence and contextualized analysis.

## Data Availability

The original contributions presented in the study are included in the article/Supplementary material, further inquiries can be directed to the corresponding author.
