# Supplementary material for: Use of artificial intelligence in building personal branding and intercultural leadership
Source: Front Artif Intell. 2026 Aug 10;9:1885655. doi: 10.3389/frai.2026.1885655 (PMC13500523; doi:10.3389/frai.2026.1885655)
Supplement: Supplementary file 1 [file Table_1.docx]

**Use of Artificial Intelligence in building personal branding and intercultural leadership**

# Sociodemographic Data

2. Gender:

[ ] Male

[ ] Female

3. Age:

4. Nationality:

5. Languages spoken:

6. Highest level of education attained:

[ ] Technical degree

[ ] Incomplete higher education

[ ] Bachelor's degree

[ ] Professional degree / Licentiate

[ ] Master's degree

[ ] Doctorate

[ ] Postdoctorate

7. Institution where you are currently pursuing or pursued higher education:

8. Academic field of study:

9. Sector of activity:

10. Role/activity:

[ ] Student

[ ] Teacher

[ ] Director/Manager

[ ] Entrepreneur

[ ] Other:

11. Years of professional experience:

12. Area or field of professional performance:

13. Size of the organization where you work:

[ ] Microenterprise

[ ] Small enterprise

[ ] Medium-sized enterprise

[ ] Large enterprise

[ ] Not working

14. Predominant work modality:

[ ] Remote

[ ] In-person

[ ] Hybrid

[ ] Not working

15. Familiarity with the use of AI:

[ ] Yes

[ ] No

16. Length of time (in years) using AI tools:

# AI Adoption and Attitudes

17. Main area of AI application:

*Carrying out assignments, class development, research, networking, etc.*

18. Have you received specific training in AI:

[ ] Yes

[ ] No

19. Perception of the usefulness of AI: *

| **Not useful at all** | 1 | 2 | 3 | 4 | 5 | **Very useful** |
| --- | --- | --- | --- | --- | --- | --- |

20. I frequently use AI tools in my studies or work.

[ ] Never

[ ] Rarely

[ ] Sometimes

[ ] Frequently

[ ] Always

21. I feel confident using AI for my professional tasks.

[ ] Never

[ ] Rarely

[ ] Sometimes

[ ] Frequently

[ ] Always

22. I have used AI for:

[ ] Content creation (texts, images, videos).

[ ] Data analysis.

[ ] Communication (translation, writing, chatbots).

[ ] Networking and contact management.

[ ] Process automation.

[ ] Other:

23. I identify the following barriers:

[ ] Lack of training or education.

[ ] High cost of tools.

[ ] Ethical or data privacy risks.

[ ] Lack of relevant data.

[ ] Internal organizational policies.

[ ] Lack of time.

[ ] Other:

24. I believe AI will improve my productivity and leadership in the next 12 months.

[ ] Strongly disagree

[ ] Disagree

[ ] Neutral

[ ] Agree

[ ] Strongly agree

25. Confidence level in the accuracy of AI-generated results.

[ ] Not reliable at all

[ ] Slightly reliable

[ ] Moderately reliable

[ ] Reliable

[ ] Very reliable

# Personal Branding

26. I maintain a consistent digital presence on professional platforms (LinkedIn, portfolio, website, etc.).

[ ] Strongly disagree

[ ] Disagree

[ ] Neutral

[ ] Agree

[ ] Strongly agree

27. My professional value proposition is clear and differentiated.

[ ] Strongly disagree

[ ] Disagree

[ ] Neutral

[ ] Agree

[ ] Strongly agree

28. I use AI tools to create, optimize, or strengthen my personal brand (designs, texts, portfolio, social media).

[ ] Never

[ ] Rarely

[ ] Sometimes

[ ] Frequently

[ ] Always

29. My personal brand is oriented toward international contexts (languages, certifications, global projection).

[ ] Never

[ ] Rarely

[ ] Sometimes

[ ] Frequently

[ ] Always

30. I actively network with professionals from other countries or international contexts.

[ ] Never

[ ] Rarely

[ ] Sometimes

[ ] Frequently

[ ] Always

31. Level of recognition of my personal brand in my professional sector.

[ ] Very low

[ ] Low

[ ] Medium

[ ] High

[ ] Very high

32. Level of consistency between my digital presence and my actual achievements/competencies.

[ ] Very low

[ ] Low

[ ] Medium

[ ] High

[ ] Very high

33. I consider that a strong personal brand directly influences my employment and leadership opportunities.

[ ] Strongly disagree

[ ] Disagree

[ ] Neutral

[ ] Agree

[ ] Strongly agree

# Intercultural Leadership

34. Mark only one oval per row.

| **Item** | **Never** | **Rarely** | **Sometimes** | **Frequently** | **Always** |
| --- | --- | --- | --- | --- | --- |
| I easily adapt to working with people from different cultures. |  |  |  |  |  |
| I listen to and value cultural perspectives different from my own. |  |  |  |  |  |
| I adjust my communication style according to the cultural context (verbal and nonverbal language). |  |  |  |  |  |
| I feel capable of leading teams with members from diverse cultures. |  |  |  |  |  |
| I use AI tools to improve my intercultural communication (translation, global interaction, networking). |  |  |  |  |  |
| I strive to understand the cultural and social norms of the teams with which I collaborate. |  |  |  |  |  |
| I promote an inclusive environment where cultural differences are respected. |  |  |  |  |  |
| I consider intercultural leadership to be a key competency for my professional development. |  |  |  |  |  |

**Validation – EFA**

**Factor analysis adequacy tests**

The preliminary adequacy tests indicate that the data are suitable for factor analysis.

- Bartlett’s Test of Sphericity: χ² = 1910.97, *p* = 7.8381e-262
- Kaiser–Meyer–Olkin (KMO) Overall Measure: 0.801

These results suggest that the correlation matrix is appropriate for factor extraction. Specifically, Bartlett’s test was statistically significant, indicating sufficient correlations among the variables, while the KMO value reflects good sampling adequacy.

**Factor loading matrix**

Factor loadings with absolute values greater than 0.40 were considered meaningful and highlighted as relevant indicators for factor interpretation.

**Explained variance**

The factor solution explained a total of 45.98% of the variance across the three extracted factors.

| **Factor** | **Explained Variance** | **Cumulative Variance** |
| --- | --- | --- |
| Factor 1 | 24.45% | 24.45% |
| Factor 2 | 12.28% | 36.73% |
| Factor 3 | 9.25% | 45.98% |

Total Explained Variance: 45.98%

Overall, the results support the adequacy of the factorial structure, with three factors accounting for a moderate proportion of the total variance.

**Factor loading matrix with promax rotation**

Table presents the factor loading matrix obtained through Promax rotation. Factor loadings with absolute values equal to or greater than 0.40 were considered statistically and theoretically meaningful for factor interpretation.

| **Variable** | **Factor 1** | **Factor 2** | **Factor 3** |
| --- | --- | --- | --- |
| Frequent_AI_Use | -0.040 | -0.026 | 0.767 |
| AI_Use_Confidence | -0.139 | 0.186 | 0.697 |
| Result_Reliability | -0.132 | 0.232 | 0.542 |
| AI_Productivity | 0.077 | -0.010 | 0.411 |
| AI_Creation_Use | 0.021 | 0.500 | 0.393 |
| AI_Intercultural_Comm | 0.501 | 0.115 | 0.378 |
| Barriers | 0.050 | 0.191 | -0.274 |
| Field_of_Use | -0.080 | 0.056 | -0.211 |
| Presence_Coherence | -0.044 | 0.723 | -0.143 |
| Platform_Presence | -0.066 | 0.507 | 0.123 |
| Networking_Global | -0.140 | 0.585 | 0.111 |
| Brand_Recognition | -0.121 | 0.757 | -0.102 |
| Adaptability | 0.851 | 0.050 | -0.078 |
| Inclusive_Environment | 0.876 | -0.014 | -0.061 |
| Brand_Opportunities | 0.073 | 0.257 | -0.059 |
| Understanding_Norms | 0.861 | 0.009 | 0.048 |
| Personal_Brand_Global | 0.101 | 0.606 | 0.047 |
| Intercultural_Leadership | 0.821 | -0.068 | 0.045 |
| Valuing_Perspectives | 0.876 | -0.083 | -0.040 |
| Communication_Style | 0.870 | -0.028 | -0.028 |
| Value_Proposition | 0.145 | 0.403 | 0.005 |
| Leadership_Capacity | 0.752 | 0.068 | 0.001 |

**Significant factor loadings**

The results show a clear three-factor structure. Each factor grouped variables that were consistent with the theoretical dimensions proposed in the study.

**Factor 1: Intercultural Leadership**

Factor 1 was mainly represented by variables related to intercultural leadership, adaptability, inclusive environments, communication, and the recognition of diverse perspectives. The highest loadings were observed in Inclusive_Environment and Valuing_Perspectives with values of 0.876, followed by Communication_Style with 0.870, and Understanding_Norms with 0.861.

The variables with significant loadings on Factor 1 were:

| **Variable** | **Loading** |
| --- | --- |
| AI_Intercultural_Comm | 0.501 |
| Adaptability | 0.851 |
| Inclusive_Environment | 0.876 |
| Understanding_Norms | 0.861 |
| Intercultural_Leadership | 0.821 |
| Valuing_Perspectives | 0.876 |
| Communication_Style | 0.870 |
| Leadership_Capacity | 0.752 |

Therefore, this factor may be interpreted as Intercultural Leadership, since it reflects the ability to communicate, adapt, lead, and value cultural diversity in global and inclusive contexts.

**Factor 2: Personal Brand and Global Visibility**

Factor 2 grouped variables associated with personal branding, digital presence, networking, brand recognition, and value proposition. The strongest loading was found in Brand_Recognition with 0.757, followed by Presence_Coherence with 0.723 and Personal_Brand_Global with 0.606.

The variables with significant loadings on Factor 2 were:

| **Variable** | **Loading** |
| --- | --- |
| AI_Creation_Use | 0.500 |
| Presence_Coherence | 0.723 |
| Platform_Presence | 0.507 |
| Networking_Global | 0.585 |
| Brand_Recognition | 0.757 |
| Personal_Brand_Global | 0.606 |
| Value_Proposition | 0.403 |

Based on these results, Factor 2 may be interpreted as Personal Brand and Global Visibility, as it captures the construction of a coherent professional identity, recognition, networking capacity, and digital positioning.

**Factor 3: AI Adoption and Attitudes**

Factor 3 was composed of variables related to the use, confidence, perceived reliability, and productivity associated with artificial intelligence. The highest loading was obtained by Frequent_AI_Use with 0.767, followed by AI_Use_Confidence with 0.697 and Result_Reliability with 0.542.

The variables with significant loadings on Factor 3 were:

| **Variable** | **Loading** |
| --- | --- |
| Frequent_AI_Use | 0.767 |
| AI_Use_Confidence | 0.697 |
| Result_Reliability | 0.542 |
| AI_Productivity | 0.411 |

Accordingly, this factor may be interpreted as AI Adoption and Attitudes, since it reflects the frequency of AI use, users’ confidence, perceived reliability of results, and the contribution of AI to productivity.

**Comparison with theoretical dimensions**

The empirical factor structure showed strong consistency with the theoretical dimensions proposed in the study. Factor 1 grouped the indicators corresponding to Intercultural Leadership, Factor 2 grouped the indicators related to Personal Brand, and Factor 3 grouped the indicators associated with AI Adoption and Attitudes.

This alignment suggests that the instrument presents an adequate factorial structure, as the items loaded primarily on the expected dimensions. In particular, the intercultural leadership dimension showed the strongest and most coherent loadings, followed by personal brand and AI adoption. Therefore, the results support the construct validity of the measurement model.
